# Five Novel Genes Related to the Pathogenesis and Progression of Pancreatic Neuroendocrine Tumors by Bioinformatics Analysis With RT-qPCR Verification

**DOI:** 10.3389/fnins.2019.00937

**Published:** 2019-09-24

**Authors:** Yu Xiao, Yuemei Yang, Yanfeng Wang, Xiaoou Li, Wenze Wang

**Affiliations:** ^1^Department of Pathology, Peking Union Medical College Hospital, Chinese Academy of Medical Sciences and Peking Union Medical College, Beijing, China; ^2^Molecular Pathology Research Center, Peking Union Medical College Hospital, Chinese Academy of Medical Sciences and Peking Union Medical College, Beijing, China; ^3^Department of Pathology, Heilongjiang Province Land Reclamation Headquarters General Hospital, Harbin, China; ^4^Department of Pathology, DaXing Hospital Affiliated to Capital Medical University, Beijing, China

**Keywords:** pancreatic neuroendocrine tumors, prognosis, biomarker, differentially expressed genes, A–D–M axis

## Abstract

**Objective:**

To explore novel related genes and potential biomarkers of pancreatic neuroendocrine tumors (PanNETs).

**Materials and Methods:**

Two data sets from ICGC and two from the NCBI GEO database were used to identify the differentially expressed genes (DEGs) in PanNETs. The common DEGs among the four sources were analyzed; furthermore, the relationship of these gene expression patterns with different PanNET grades, their mutation status and corresponding impact on prognosis, the interaction network, and the relationship with three known PanNET genes (ATRX, DAXX, and MEN1) were analyzed by two other GEO data and cBioPortal database. Finally, the expressions of novel DEGs were validated in Chinese PanNET tissues by RT-qPCR.

**Results:**

Five new DEGs (ABCC8, PCSK2, IL13RA2, KLKB1, and PART1) and one confirmed DEG-ISL1 were identified. The mutation counts of DEGs increased with the tumor grade increasing from G1 to G3, and PanNET patients present vascular invasion or are deceased. These DEG expression patterns in PanNETs are quite different from that of pancreatic ductal adenocarcinoma and are related to A–D–M (ATRX–DAXX–MEN1) mutation. ABCC8 and KLKB1 are co-occurrence with the A–D–M axis in PanNETs. Importantly, patients with DEG mutations have a lower survival rate. RT-qPCR verification results of KLKB1 (*P* < 0.01), IL13RA2 (*P* < 0.01), ABCC8 (*P* < 0.01), and PART1 (*P* < 0.0001) expressions in Chinese PanNET tissues are consistent with our database analysis, which were significantly up-regulated. However, the expression of PCSK2 (*P* < 0.01) was contrary to our bioinformatics analysis, which was significantly down-regulated, suggesting that the expression trend of PCSK2 may be different among different races. These results indicated that these five genes may play an important role in the occurrence and progression of PanNETs.

**Conclusion:**

Five novel common DEGs identified are related to the development and prognosis of PanNETs and may serve as specific biomarkers and therapeutic targets.

## Introduction

Pancreatic neuroendocrine tumors originated from the cells of the endocrine part within the pancreas. Most PanNETs are benign, and once termed “islet cell tumors,” while aggressive PanNETs had traditionally been termed “islet cell carcinoma.” PanNETs are a relatively rare but clinically important form of pancreatic neoplasia, and quite distinct from the usual pancreatic neoplasia. Only 2–5% of clinically significant pancreas neoplasia are PanNETs (Anderson and Bennett, 2016). For the WHO classification of tumors of endocrine organs, in addition to the criterion of their mitotic count and the proliferation index with Ki-67 expression, morphological features have been added (Lloyd et al., 2017).

In recent years, the incidence of PanNETs has increased (Anderson and Bennett, 2016). Some PanNETs are functional and cause symptoms related to excessive secretion of hormones or active polypeptides. However, up to 60% of PanNETs are non-functional. There is no secretion, or the quantity or type of products does not cause a clinical symptom, although blood levels may be elevated (Jensen et al., 2010). In total, about 85% of PanNETs have elevated blood hormone markers (Burns and Edil, 2012).

There are several markers found to be related to PanNETs, such as ATRX/DAXX and MEN1. A study screened the most commonly mutated genes in 68 PanNETs and found that 44% of the tumors had somatic inactivating mutations in MEN-1, which encodes menin, a component of a histone methyltransferase complex, and 43% had mutations in genes encoding either of the two subunits of a transcription/chromatin remodeling complex consisting of Jiao et al. (2011). Clinically, mutations in the MEN1 and DAXX/ATRX genes were associated with better prognosis. Other researches have similar results (Park et al., 2017; Vandenbussche et al., 2017). However, more related genes are needed to help us predict PanNET behavior and understand the potential mechanism.

In this study, we explored novel related genes and potential biomarkers for PanNETs through all databases attained online using a bioinformatic method and analyzed their expression, mutation, and effect on survival of PanNETs. Finally, we validated the novel selected genes in Chinese Pan NETs patients.

## Materials and Methods

### Sample Collection

Our samples were collected from the ICGC database^1^ and the NCBI GEO database.^2^ There are four sources in these databases: GSE73338 and GSE117851 are from NCBI GEO database, and PanNETs-Italy (PAEN-IT) and PanNETs-Austria (PAEN-AU) are from ICGC database. GSE73338 contains 97 samples: normal pancreas, 5 samples; normal pancreas islet, 4 samples; PanNETs, 81 samples; Met-NETs, 7 samples. GSE117851 contains 47 samples: A–D–M (ATRX–DAXX–MEN1) Mutant PanNETs, 30 samples; A–D–M WT PanNETs, 17 samples (details of GSE73338 and GSE117851 in Supplementary Material 1). Pancreatic endocrine neoplasms coexpression genes (PAEN Cogene) contains 104 samples of Italian (PAEN-IT) and Austrian (PAEN-AU) patients. More details are shown in Table 1.

**TABLE 1:** Sample details in PAEN Cogene in the ICGC database.

	**Female**	**Male**	**Age**	**Primary site**	**Disease status**	
					**Stable**	**Progression**
PAEN-IT	13	24	55.1 ± 13.3	Pancreas	16	21
PAEN-AU	28	39	57.0 ± 14.9	Pancreas	52	15

Seventeen samples from fresh tumor tissues and eight samples from paired adjacent non-tumor tissues were collected from PanNET patients after surgical resection at the Peking Union Medical College Hospital, Beijing, China. Histopathology of all tissues was evaluated on hematoxylin and eosin-stained sections by an experienced gastrointestinal-hepato-pancreatobiliary pathologist to ensure the nature of the tissue, greater than 80% tumor cellularity, and absence of necrosis. This study was carried out in accordance with the guidelines of the Ethics Committee of Peking Union Medical College Hospital, with written informed consent from all subjects. All subjects gave written informed consent in accordance with the Declaration of Helsinki. The protocol was approved by the Ethics Committee of Peking Union Medical College Hospital.

### Differently Expressed Genes Identification

The DEGs between different samples were analyzed using GEO2R.^3^ For GSE73338, no mutations were identified in 33 samples, while mutations in ATRX, DAXX, MEN1, ATM, TSC2, and PTEN were identified in 64 samples. These gene mutant samples were compared with wild-type samples (2015 Mut vs. WT). On the other hand, differences in clinical symptoms are also used for comparison. Normal group contains normal pancreas samples (2011) and normal pancreas islet samples (Burns and Edil, 2012), and the PAEN group contains PanNET samples (81 primary and 7 metastatic, PAEN vs. Normal).

In GSE117851, A–D–M (ATRX–DAXX–MEN1) mutant samples were compared with A–D–M wild-type samples (2018 Mut vs. WT). In PAEN Cogene, samples from Italy patients were compared with that from Austrian patients (PAEN-IT vs. PAEN-AU). The number of DEGs was counted separately. The common DEGs among these PanNET sources were analyzed by venny 2.1^4^ and jvenn.^5^ These common DEGs were preliminarily analyzed in database. Perhaps racial differences have brought about many changes in genetic backgrounds that are not related to PanNETs.

### Expression Patterns Analysis for Common DEGs

Expression patterns in different tissues and gene-mutation tissue may suggest the pathways that the target involved in PanNETs. GSE73338, GSE43797, GSE117851, and GSE118014 were used to analyze expression patterns for six common DEGs. In GSE73338, we compared the expression in normal tissue, PanNETs, and PCA. In GSE43797, the expression in normal tissue, PanNETs, and Met-NETs was compared. In GSE117851 and GSE118014, A–D–M mutant samples were compared with A–D–M wild-type samples for six common DEGs.

### Prognostic Analysis for Common DEGs

We analyzed the relationship between identified DEGs and the prognosis (overall survival and disease free survival) using ICGC databases. The donors with mutation gene and donors with wild-type gene were compared.

### Gene Mutation Analysis in cBioPortal Database

cBioPortal database^6^ was used to collect the data of PanNET cases. A study about whole-genome landscape sequencing of PanNETs was chosen (Scarpa et al., 2017). In this study, 98 primary PanNETs were collected. These clinically sporadic PanNETs contained higher-than-expected proportion of germline mutations, including previously unreported mutations. The mutation counts of the identified DEGs (including ARTX, DAXX, and MEN1 additionally) were statistically compared between function and non-function PanNET cases, different WHO grade groups, with and without vascular invasion group, and overall patient survival status.

### Analysis of Gene Interaction Network

The co-occurrence/mutual exclusivity analysis of identified common DEGs with ATRX, DAXX, and MEN1 were performed in our study. The cases present neither, or one or two kinds of gene mutations were counted and used to evaluate the relevance, and we plotted the DEG interaction networks that contain the identified common DEGs and the 50 most frequently altered neighbor genes (out of a total of 334).

### RNA Extraction and RT-qPCR

Total RNA was extracted using the Trizol reagent (Invitrogen, United States) according to the manufacturer’s instructions. The cDNA was synthesized by using the SuperScript III Reverse Transcriptase Kit (Invitrogen, United States). RT-qPCR was performed with Power SYBR Green PCR Master Mix (TransGen Biotech, China) on the ABI 7500 fast real-time PCR system. The amplification reaction procedure was as follows: 95°C for 10 min, followed by 95°C for 15 s and 60°C for 1 min for 40 cycles. GAPDH was applied as internal control for mRNA, and the relative expression level of mRNA was calculated by relative quantification (2^–ΔΔCT^) method. Primer sequences are listed in Table 2.

**TABLE 2:** The primers used for RT-qPCR with their sequence.

**Gene**	**Forward primer**	**Reverse primer**
KLKB1	TGCGTTCTCAGATGTGGAT GTTG	TGAGGAGTAGAGGAACTT GGTGTG
IL13RA2	AACCTGGCATAGGTGTACT TCTTG	CACACTGTAATGCATGATC CAAGC
ABCC8	CAGGATGAGGAAGAGGAGG AAGAG	GGCGGAGGACAGGTAC TTGG
PART1	CATCCAAGGCCGTGTCAGA ACTC	GCTAAGTGATTGGCTGGCT CTGG
PCSK2	CACTGGCTCTGGAGGCT AACC	ACTGATGGACCTCGTCGT GAAG
GAPDH	GGCAGTGATGGCATGG ACTGT	CCTTCATTGACCTCAAC TACA

### Statistical Analysis

All data are presented as means ± standard deviation of three independent experiments. Two group comparisons were analyzed by the two-sided Student *t* test. Tukey’s multiple comparison test was used to analyze the variance of the data and to estimate the level of significance. *P* < 0.05 was considered significant.

## Results

### Five Novel Common DEGs Were Identified Among Four PanNET Sources

Eighty-one DEGs were identified between 2015 Mut vs. WT. More DEGs (2145 genes) were identified between PAEN vs. Normal of GSE73338. This result suggests less different gene expression related to different gene mutation status than PanNET vs. Normal tissues. A total of 807 DEGs mutations were identified between the A–D–M mutant and the A–D–M wild-type group, while 12,109 DEG mutations were identified between Italian and Austrian patients.

**FIGURE 1:**
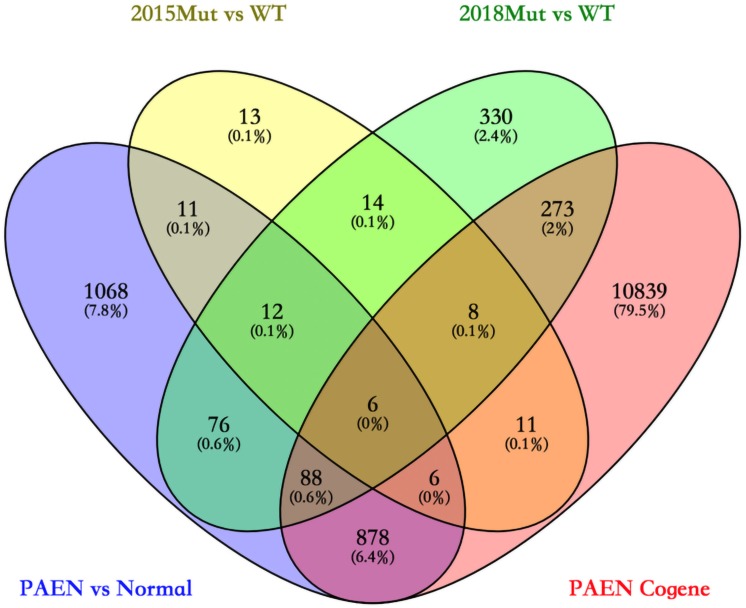
Venn diagram among four data sets in ICGC and GEO databases. 2015 Mut vs. WT means the gene mutant samples comparing with wild-type samples in the GSE73338 database. 2018 Mut vs. WT means the gene mutant samples compared with wild-type samples in the GSE117851 database.

We focused on the common DEGs among different sources. In this study, six of these genes were identified shown in a Venn diagram (Figure 1). ABCC8, PCSK2, IL13RA2, ISL1, and KLKB1 are protein coding and PART1 is lincRNA. ISL1 was a previously reported PanNET-related gene, while the other five were newly found genes. Mutations of DEGs have been verified in all diseases (Table 3).

**TABLE 3:** Details of six common DEGs.

**Symbol**	**Name**	**Location**	**Type**	**#Donors affected**		**#Mutations**
				**Total**	**Across all projects**	
ABCC8	ATP-binding cassette, sub-family C (CFTR/MRP), member 8	Chr11:17414432-17498449	Protein coding	1319/15,110 (8.73%)	1319/15,285 (8.63%)	2681
PART1	Prostate androgen-regulated transcript 1 (non-protein coding)	Chr5:59783540-59843484	LincRNA	751/15,153 (4.96%)	751/15,285 (4.91%)	1172
PCSK2	Proprotein convertase subtilisin/kexin type 2	Chr20:17206752-17465223	Protein coding	2137/15,110 (14.14%)	2137/15,285 (13.98%)	5792
IL13RA2	Interleukin 13 receptor, alpha 2	ChrX:114238538-114254540	Protein coding	487/14,927 (3.26%)	487/15,285 (3.19%)	630
ISL1	ISL LIM homeobox 1	Chr5:50678921-50690564	Protein coding	607/15,110 (4.02%)	607/15,285 (3.97%)	825
KLKB1	Kallikrein B, plasma (Fletcher factor) 1	Chr4:187130133-187179625	Protein coding	938/14,927 (6.28%)	938/15,285 (6.14%)	1517

### The DEGs Expression in PanNETs Is Quite Different From PCA

**FIGURE 2:**
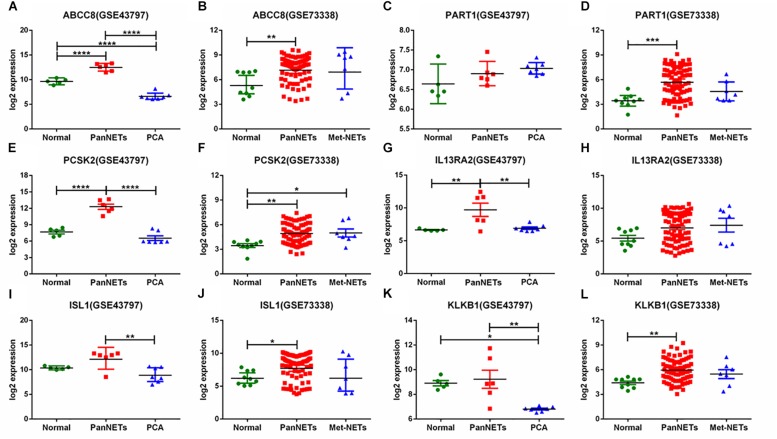
Six gene expression levels in PanNETs, PCA, and normal pancreatic tissues. **(A,C,E,G,I,K)** are the expression levels of six genes in GSE43797. **(B,D,F,H,J,L)** are the expression levels of six genes in GSE73338. PCA = ductal adenocarcinoma, PanNETs = pancreatic neuroendocrine tumors, Met-NETs = metastases from pancreatic neuroendocrine tumors. ^∗^*P* <c 0.05, ^∗∗^*P* < 0.01, ^∗∗∗^*P* < 0.001, ^****^P < 0.0001.

In GSE43797, the DEG expression of PanNET samples is quite different from that of PCA (*P* < 0.001) except the lincRNA-PART1 (Figure 2). This result is consistent with previous studies that PanNETs are quite distinct from the usual pancreatic neoplasia. However, in GSE73338, there were no different expressions between primary and metastatic PanNETs, which suggests that these common DEGs are not involved in the metastasis of PanNETs. The expression mode between normal tissue and PanNET tissue varied in GSE73338 and GSE43797. ABCC8 and PCSK2 were found to be differentially expressed between normal tissue and PanNET tissue in GSE73338 and GSE43797, but the results of the other four genes were contradictory. It is possible that the difference arose from different ethnic, region, or detection methods. The GSE73338 database contains primary and metastatic PanNETs, while the GSE43797 database contains only the former. All the gene expression varied largely among PanNET samples compared to other samples, which suggested that PanNETs contain many subtypes with different expression patterns.

### Gene Expression Is Probably Related to A–D–M Mutation

**FIGURE 3:**
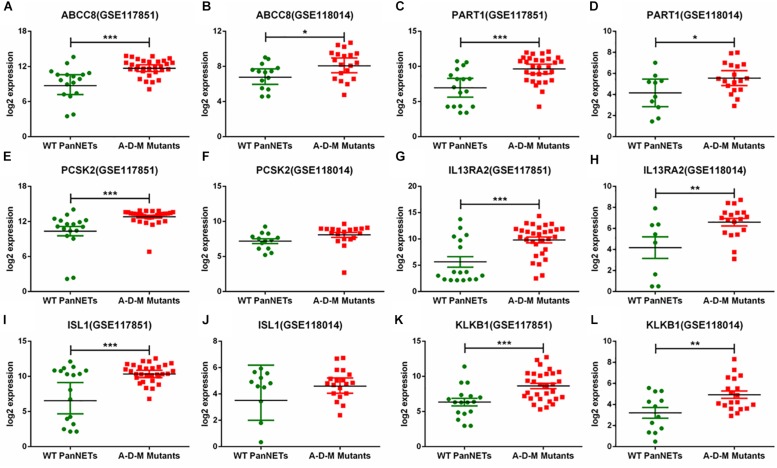
Six gene expression levels in A–D–M mutant and A–D–M wild-type PanNETs. **(A,C,E,G,I,K)** are the expression levels of six genes in GSE117851. **(B,D,F,H,J,L)** are the expression levels of six genes in GSE118014. ^∗^*P* < 0.05, ^∗∗^*P* < 0.01, ^∗∗∗^*P* < 0.001.

In GSE117851 and GSE118014, A–D–M mutant samples were compared with A–D–M wild-type samples for six common DEGs, but the results of comparison of some genes are different (Figure 3). In GSE117851, all the six gene expression changed in A–D–M mutant samples (*P* < 0.0001). However, in GSE118014, expression changes were lighter than in GSE117851, and no significant expression changes are identified in PCSK2 and ISL1. For all DEGs, the expression in A–D–M mutant samples is up-regulated compared with WT samples. A–D–M mutation was observed in a large number of PanNET cases and involved in mTOR pathways. The six DEGs identified in our study may also be activated by the same pathway.

### Patients With Gene Mutations Have a Low Survival Rate

**FIGURE 4:**
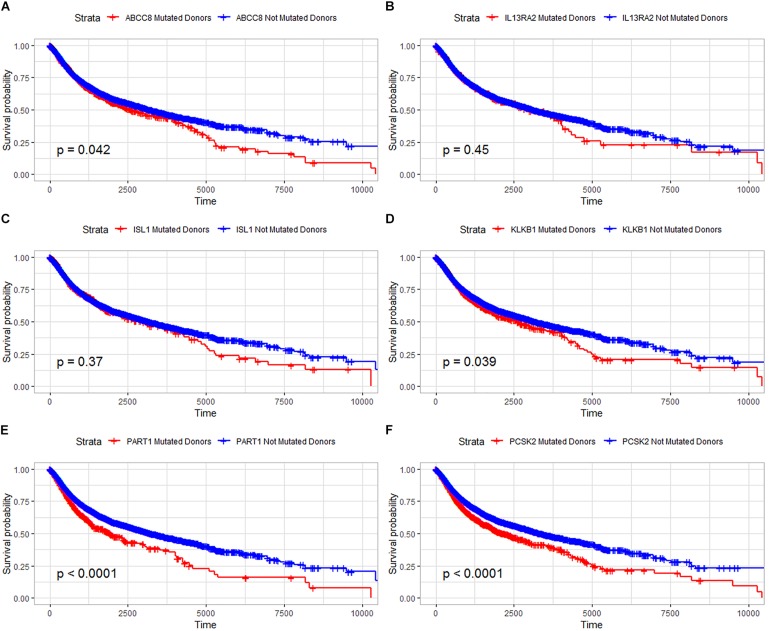
Overall survival analysis for six common DEGs in all diseases from the ICGC database. **(A)** Overall survival analysis for ABCC8, **(B)** overall survival analysis for IL13RA2, **(C)** overall survival analysis for ISL1, **(D)** overall survival analysis for KLKB1, **(E)** overall survival analysis for PART1, and **(F)** overall survival analysis for PCSK2.

In our study, the trend of overall survival and disease-free survival was similar (Figure 4). The prognosis analysis is different with A–D–M mutation. Combined with the result in Figure 3, A–D–M mutation related to up-regulation in six DEGs. Up-regulation of six DEGs is related to better prognosis, while the mutation of six DEGs may decrease the expression and may be involved in the poor survival of PanNETs. The above results suggested that these six genes may play an important role in the pathogenesis and progression of PanNETs.

### Gene Mutations of ABCC8, KLKB1, and IL13RA2 Are Rare in PanNET Cases

**FIGURE 5:**
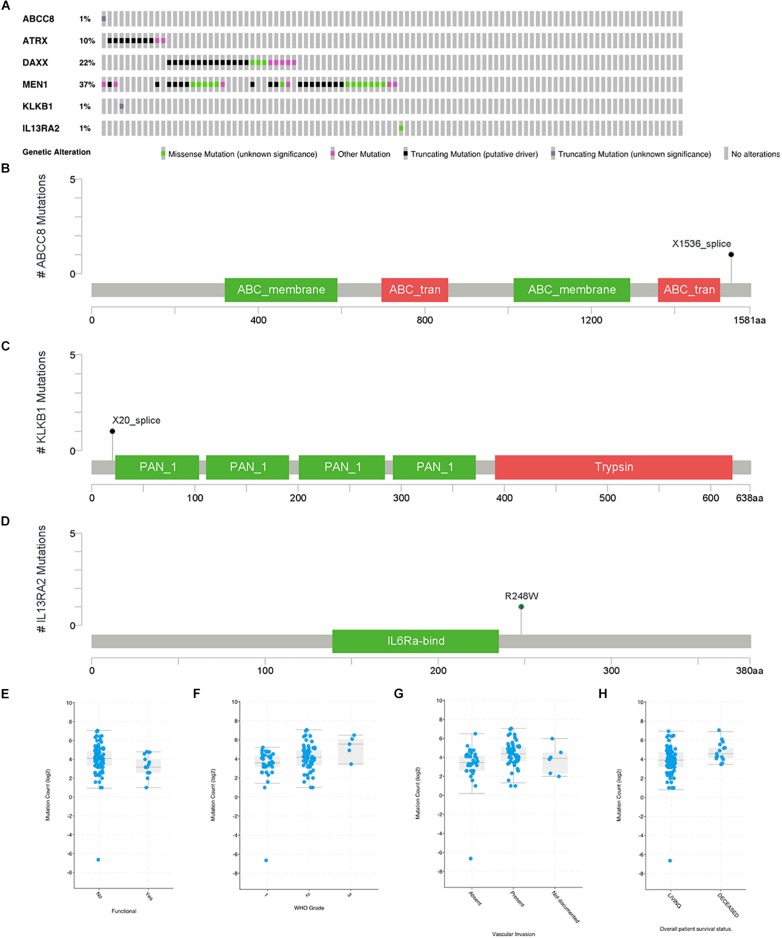
Mutations of ABCC8, KLKB1, IL13RA2, ATRX, DAXX, and MEN1 in 98 primary PanNET cases. WHO Grade = World Health Organization Grade. **(A)** Mutation statistics for ABCC8, KLKB1, IL13RA2, ATRX, DAXX and MEN1; **(B)** the location of mutation in ABCC8; **(C)** the location of mutation in KLKB1; **(D)** the location of mutation in IL13RA2; **(E)** comparison of mutation count between function PanNETs and non-function PanNETs; **(F)** comparison of mutation count among different WHO grades; **(G)** comparison of mutation count among different vascular invasion state; and **(H)** comparison of mutation count between alive and deceased patients.

In a study on 98 primary PanNETs, mutations were found in one case for ABCC8, KLKB1, and IL13RA2 gene (Figure 5A). MEN1 mutations were found in 37% of the cases; DAXX, 22%; and ATRX, 10%. For ABCC8, X1536 splice was identified (Figure 5B), while for KLKB1, X20 splice was found (Figure 5C). These two mutations are truncating mutation. In IL12RA2, there was a missense mutation (R248W) (Figure 5D). For ARTX, eight cases are truncating mutations and two cases are other mutations. For DAXX, there were 13 truncating mutations, 5 other mutations, and 3 missense mutations. For MEN1, there were 17 truncating mutations, 6 other mutations, and 13 missense mutations. PCSK2, ISL1, and PART1 mutations were not found in this study. Gene mutations of six common genes that we identified were rare in 98 primary PanNET cases.

There were more mutations in non-functional PanNETs than in functional PanNETs (Figure 5E). The mutation counts increased with the disease grade progressing from G1 to G3 (Figure 5F), and PanNET patients present vascular invasion (Figure 5G) or are deceased (Figure 5H). These results were consistent with previous studies.

### ABCC8-MEN1 and ATRX-KLKB1 Are Co-occurrence Gene Pairs

In this co-occurrence/mutual exclusivity analysis, we identified three co-occurrence pairs among ABCC8, KLKB1, IL13RA2, ATRX, DAXX, and MEN1 (Table 4). DAXX–MEN1 had been confirmed in other studies. We first identified ABCC8–MEN1 and ATRX–KLKB1 as co-occurrence in PanNETs. This result suggests that ABCC8 and KLKB1 may be involved in PanNETs through the A–D–M axis because these two gene mutations occurred in the same case.

**TABLE 4:** Co-occurrence/mutual exclusivity analysis among ABCC8, KLKB1, IL13RA2, and 3 PanNET-related genes.

**Gene A**	**Gene B**	**Neither**	**A Not B**	**B Not A**	**Both**	**Log odds ratio**	***P*-value**	**Adjusted *P*-value**	**Tendency**
DAXX	MEN1	55	7	21	15	1.725	<0.001	0.001	Co-occurrence
ATRX	DAXX	66	10	22	0	<−3	0.068	1	Mutual exclusivity
ATRX	KLKB1	88	9	0	1	>3	0.102	1	Co-occurrence
ABCC8	MEN1	62	0	35	1	>3	0.367	1	Co-occurrence
ATRX	MEN1	55	7	33	3	−0.336	0.463	1	Mutual exclusivity
MEN1	KLKB1	61	36	1	0	<−3	0.633	1	Mutual exclusivity
MEN1	IL13RA2	61	36	1	0	<−3	0.633	1	Mutual exclusivity
ABCC8	DAXX	75	1	22	0	<−3	0.776	1	Mutual exclusivity
DAXX	KLKB1	75	22	1	0	<−3	0.776	1	Mutual exclusivity
DAXX	IL13RA2	75	22	1	0	<−3	0.776	1	Mutual exclusivity
ABCC8	ATRX	87	1	10	0	<−3	0.898	1	Mutual exclusivity
ATRX	IL13RA2	87	10	1	0	<−3	0.898	1	Mutual exclusivity
ABCC8	KLKB1	96	1	1	0	<−3	0.99	1	Mutual exclusivity
ABCC8	IL13RA2	96	1	1	0	<−3	0.99	1	Mutual exclusivity
KLKB1	IL13RA2	96	1	1	0	<−3	0.99	1	Mutual exclusivity

**FIGURE 6:**
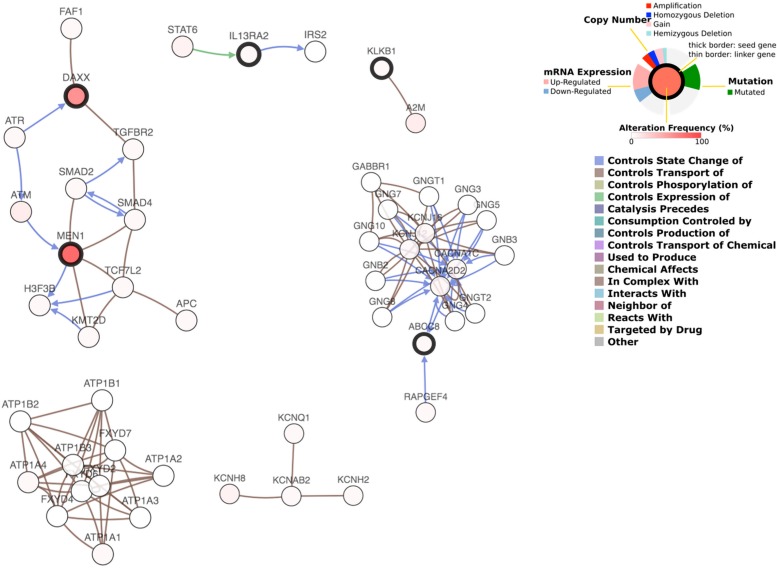
Gene interaction network plot.

The predicted interaction between 6 genes and the 50 most frequently altered neighbor genes are shown in Figure 6. The transcription factor STAT6 was predicted to regulate the expression of IL13RA2, while IL13RA2 changes the state of IRS2, an insulin receptor substrate. ABCC8 was supposed to interact with many G-protein subunits and may be involved in signal transduction in PanNETs. KLKB1 interacts with A2M, a protease inhibitor and cytokine transporter. A2M inhibits a broad spectrum of proteases, including trypsin, thrombin, and collagenase. It can also inhibit inflammatory cytokines and thus disrupts inflammatory cascades. KLKB1 is involved in protease inhibition and inflammatory cascades, and play a role in cancer progression, while the underlying mechanism needs further research.

### RT-qPCR Validation of Five DEGs in Chinese PanNETs

**FIGURE 7:**
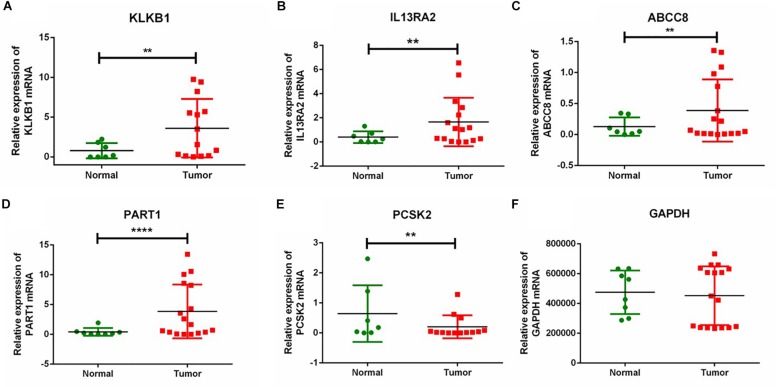
RT-qPCR validation of five DEGs in Chinese PanNETs. **(A)** Expression level of KLKB1 gene, **(B)** expression level of IL13RA2 gene, **(C)** expression level of ABCC8 gene, **(D)** expression level of PART1 gene, **(E)** expression level of PCSK2 gene, and **(F)** expression level of GAPDH gene. ^∗∗^*P* < 0.01, ^****^P < 0.0001.

To further verify the expression of the five novel DEGs in PanNET tissues, we detected their expression in 17 samples of tumor tissue from Chinese PanNETs and 8 samples of adjacent non-tumor tissues. The results showed that the expression of KLKB1 (*P* < 0.01), IL13RA2 (*P* < 0.01), ABCC8 (*P* < 0.01), and PART1 (*P* < 0.0001) was significantly up-regulated, while PCSK2 (*P* < 0.01) was significantly down-regulated in PanNET tissues compared to the non-tumor tissues (Figure 7 and Supplementary Material 2).

## Discussion

In our study, we collected 248 PanNET samples from four database sources: GSE73338, GSE117851, PAEN-IT, and PAEN-AU. Five novel PanNET-related genes and one previous reported gene were identified among four database sources. The gene expression patterns of PanNETs are quite different from PCA through analyzing the expression of the six DEGs in GSE73338 and GSE43797. Meanwhile, gene expression varied vastly in PanNET samples, which suggested that PanNETs may contain many subtypes with different gene phenotypes. Interestingly, the expressions of the six common DEGs in A–D–M mutant samples were all up-regulated compared with WT samples, indicating that the six common DEGs probably related to the three known PanNET genes. Further, cBioPortal analysis results supported this conclusion. The six up-regulated genes identified in our study may also be activated by the mTOR pathway. Patients with mutations of six common DEGs have a lower survival rate. Therefore, we predicted that the five new genes may play an important role in the progression and prognosis of PanNETs.

ABCC8 is a member of the superfamily of ATP-binding cassette (ABC) transporters. ABC proteins transport various molecules across extra- and intra-cellular membranes. ABCC8 regulated K+ channel, and ABCC8/KIR6.2 channels found in insulin-secreting pancreatic beta cells are the cause of monogenic forms of hyperinsulinemic hypoglycemia and neonatal diabetes (Bryan et al., 2007). Many mutations in ABCC8 render the channel unable to traffic to the cell surface, thereby reducing channel function (Yan et al., 2007). In this study, we first identified ABCC8 to be related to PanNETs, but changes in K+ channel core-KIR6.2 protein was not observed in PanNETs. It is possible that ABCC8 is related to MEN1 and involved in PanNETs, because the two genes are co-occurrence in our study.

Kallikrein B1 encodes a glycoprotein that participates in the surface-dependent activation of blood coagulation, fibrinolysis, kinin generation, and inflammation. KLKB1 mRNA is significantly higher in CLL patients than in healthy blood donors and is associated with an increased risk for CLL and will serve as a novel biomarker (2015) (Adamopoulos et al., 2015). In our study, KLKB1 interacts with A2M. In 2015, researchers identified a novel A2M-ALK rearrangement in lung interstitial tumor (Onoda et al., 2014). A2M mutations are also found in cBioPortal PanNET cases (data not displayed). It could be that KLKB1 is affected by its co-occurrence gene-ATRX. PanNETs frequently use the ALT pathway for telomere maintenance, which is strongly correlated with ATRX and DAXX (Vandenbussche et al., 2017).

IL13RA2 is a subunit of the interleukin 13 (IL-13) receptor complex. IL13RA2 is overexpressed in the majority of high-grade astrocytomas and other tumors, such as renal cell carcinoma, brain tumor, and ovarian cancer (Wu et al., 2005; Kioi et al., 2006; Kim et al., 2015; Noboru et al., 2015). It has been validated to be a target for therapeutic applications. IL-13 enhances the expression of EMT-promoting factor ZEB1, and STAT6 knockdown significantly reversed IL-13-induced EMT (Cao et al., 2016). IL13RA2 may be involved in the IL-13/STAT6 axis and regulates the EMT process in PanNETs. In pancreatic cancer cell lines, histone deacetylation inhibition increases IL13RA2 expression (Fujisawa et al., 2011). Part of our finding is consistent with this study.

Islet 1 expression is a reliable marker for PanNETs and their metastases (Schmitt et al., 2008; Graham et al., 2013). It is a transcription factor and regulates CCNB1, CCNB2, and c-MYC genes (Shi et al., 2016). The single-nucleotide polymorphism at the PCSK2 gene and the genetic predisposition are related to the diagnosis of T2DM in Chinese (Zheng et al., 2014). lncRNA-PART1 functions as a ceRNA and may serve as a therapeutic target for ESCC patients. STAT1 can bind to the promoter region of lncRNA PART1, resulting in its activation. Activated PART1 promoted gefitinib resistance by competitively binding to miR-129 to facilitate Bcl-2 expression in ESCC cells (Min et al., 2018). Part of these six DEGs’ mechanism in other tumors was researched, but their mechanism in PanNETs is still unknown.

We further validated the expression of the five novel DEGs in Chinese PanNETs. Our results indicated that the expression of KLKB1 (*P* < 0.01), IL13RA2 (*P* < 0.01), ABCC8 (*P* < 0.01), and PART1 (*P* < 0.0001) was consistent with our bioinformatics analysis, which was significantly up-regulated in PanNETs compared with the non-tumor tissues. However, the expression of PCSK2 (*P* < 0.01) was contrary to our bioinformatics analysis, which was significantly down-regulated in PanNETs (Figure 7). The results suggested that the expression trend of PCSK2 may be different among different races.

The limitations of this study are as follows: first, three common DEGs: PSCK2, ISL1, and lncRNA-PART1 mutations were not analyzed because of data limitations; second, the mechanism of these new identified genes was needed to be researched in further studies.

In summary, we identified four new PanNET-related genes and 1 lncRNA-PART1 using the ICGC database and the NCBI GEO database. The analysis results of the five new common DEGs in GSE73338 and GSE43797 show that gene expression patterns of PanNETs are quite different from PCA. With the increase of malignant grade of PanNETs, the mutation counts increased. Importantly, patients with mutations of these five novel common DEGs have a lower survival rate. Our results suggested that the five new genes may play an important role in the development and prognosis of PanNETs. Furthermore, in our study, we found that ABCC8–MEN1 and ATRX–KLKB1 were co-occurrence in PanNETs, while ABCC8, KLKB1, and IL13RA2 were mutually exclusive with ATRX, DAXX, and MEN1. These results will be helpful to further study the molecular mechanism of PanNETs.

## Data Availability

Publicly available datasets were analyzed in this study. This data can be found here: https://dcc.icgc.org/.

## Ethics Statement

This study was carried out in accordance with the guidelines of the Ethics Committee of Peking Union Medical College Hospital, with written informed consent from all subjects. All subjects gave written informed consent in accordance with the Declaration of Helsinki. The protocol was approved by the Ethics Committee of Peking Union Medical College Hospital.

## Author Contributions

YX and YY contributed to the preparation of the research, literature review, and writing of the manuscript. YW and XL collected the data. WW and YX provided ideas and recommendations, and reviewed the manuscript. All authors have seen and agreed with the contents of the manuscript.

## Conflict of Interest Statement

The authors declare that the research was conducted in the absence of any commercial or financial relationships that could be construed as a potential conflict of interest.

## Footnotes

1 https://dcc.icgc.org/

2 https://www.ncbi.nlm.nih.gov/geo/

3 http://www.ncbi.nlm.nih.gov/geo/geo2r

4 http://bioinfogp.cnb.csic.es/tools/venny/index.html

5 http://jvenn.toulouse.inra.fr/app/example.html

6 http://www.cbioportal.org/

## Abbreviations

ABCC8: ATP binding cassette subfamily C member 8
ALT: alternative lengthening of telomeres
ATRX: alpha thalassemia/mental retardation syndrome X-linked
ATRX–DAXX–MEN1: A–D–M
CLL: chronic lymphocytic leukemia
DAXX: death-domain associated protein
DEGs: differentially expressed genes
ESCC: esophageal squamous cell carcinoma
IL13RA2: interleukin 13 receptor subunit alpha 2
ISL1: Islet 1
KLKB1: Kallikrein B1
Met-NETs: metastases from pancreatic neuroendocrine tumors
PanNETs: pancreatic neuroendocrine tumors
PCA: pancreatic ductal adenocarcinoma
T2DM: type 2 diabetes
WHO: World Health Organization.
