# Supplementary material for: Five Novel Genes Related to the Pathogenesis and Progression of Pancreatic Neuroendocrine Tumors by Bioinformatics Analysis With RT-qPCR Verification
Source: Front Neurosci. 2019 Sep 24;13:937. doi: 10.3389/fnins.2019.00937 (PMC6771308; doi:10.3389/fnins.2019.00937)

Supplementary Material 2:Dissolution Curve of RT-qPCR Experimental Genes.

GAPDH

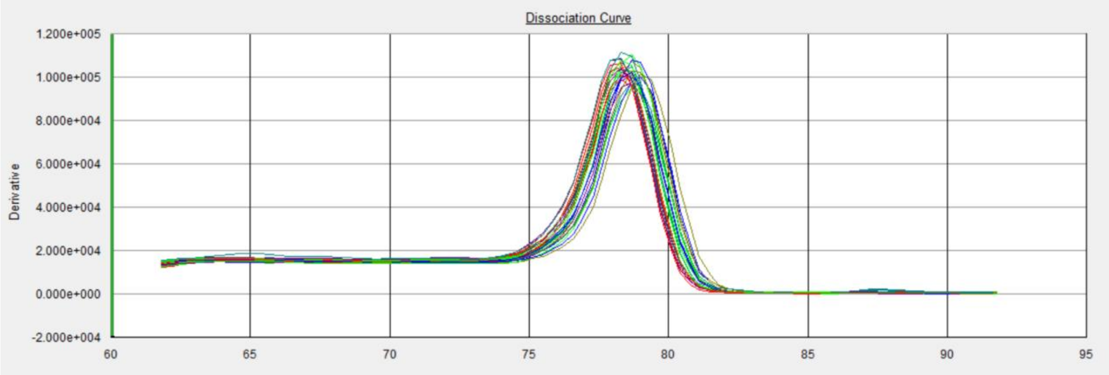

KLKB1

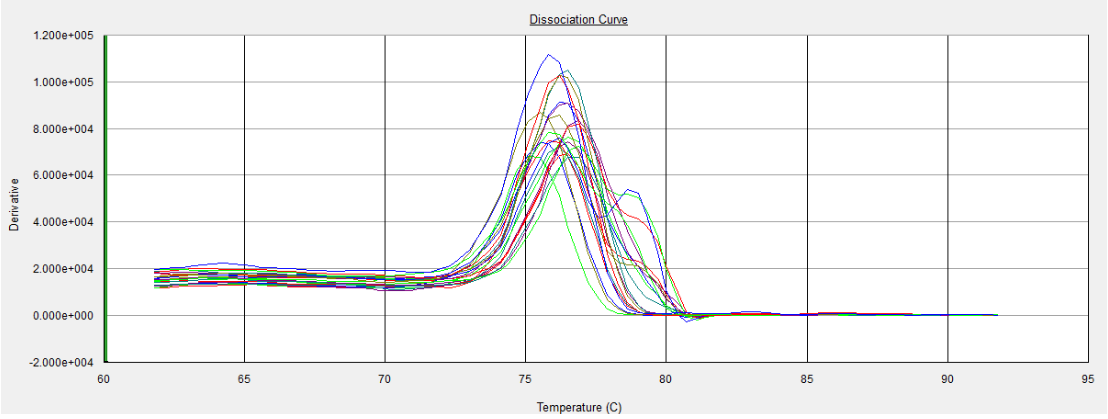

IL13RA2

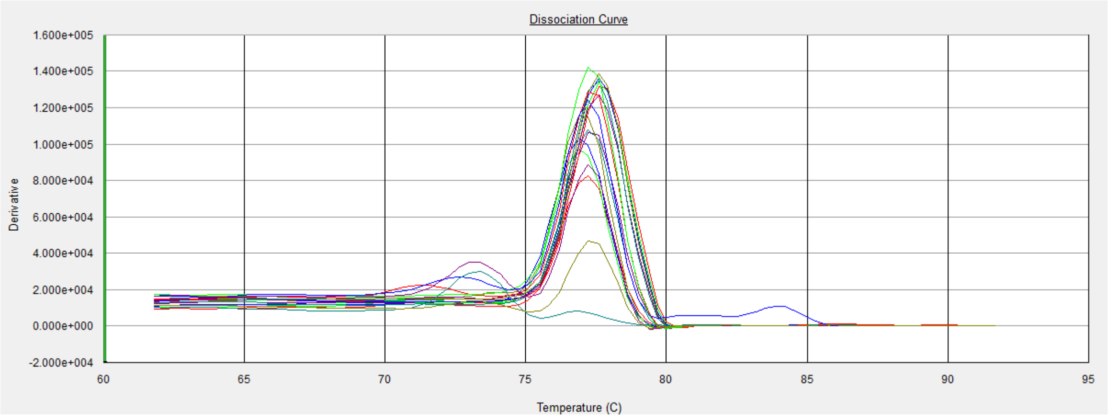

PCSK2

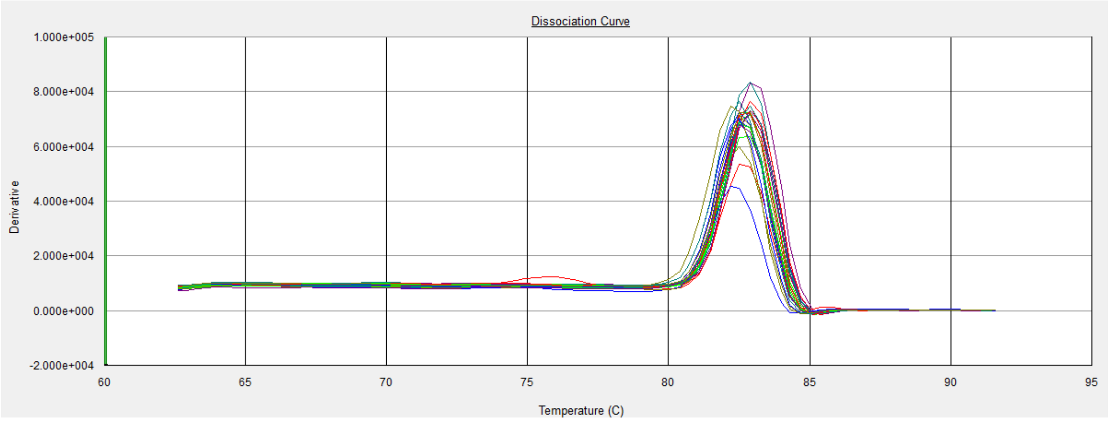

ABCC8

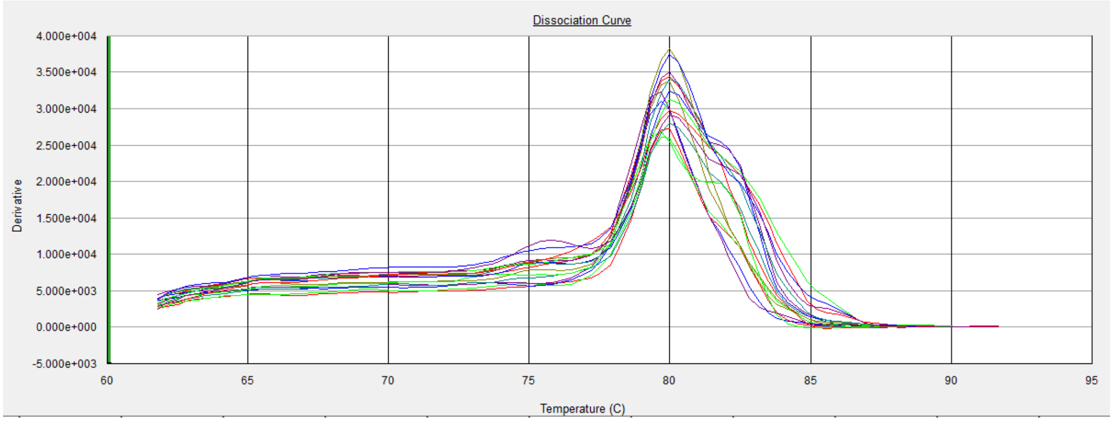

PART1

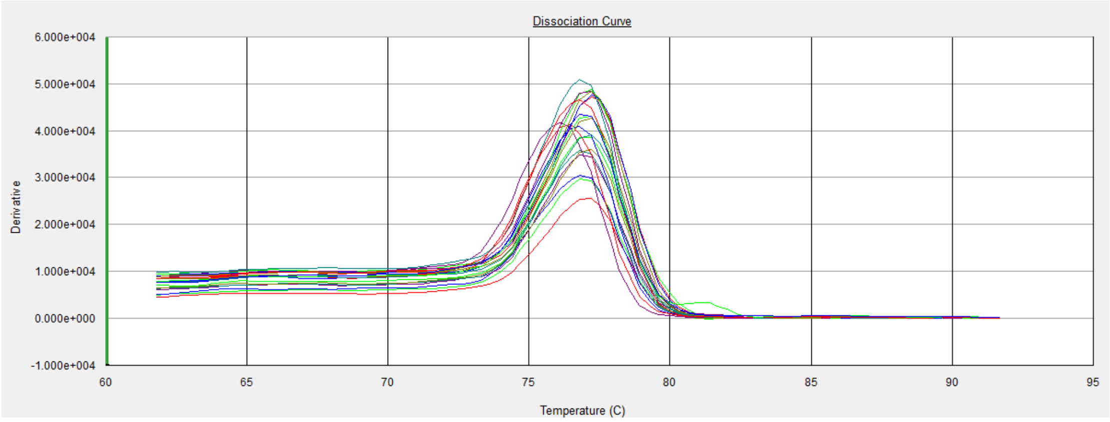

Supplement: Supplementary file 2 [file Image_1.pdf]
